# The Impact of Emotional Leadership on Subordinates' Job Performance: Mediation of Positive Emotions and Moderation of Susceptibility to Positive Emotions

**DOI:** 10.3389/fpsyg.2022.917287

**Published:** 2022-06-24

**Authors:** Jin Wan, Kun ting Pan, Yuan Peng, Ling qiang Meng

**Affiliations:** ^1^School of Economics and Management, East China Jiaotong University, Nanchang, China; ^2^Research Centre for High-Speed Railway and Regional Development, East China Jiaotong University, Nanchang, China; ^3^School of Computer and Information Engineering, Jiangxi Agricultural University, Nanchang, China; ^4^School of Economics and Management, University of Chinese Academy of Sciences, Beijing, China

**Keywords:** emotional leadership, positive emotions, susceptibility to positive emotions, job performance, affective events theory

## Abstract

Employees' emotions have an important effect on their job performance, thus leaders can influence subordinates' emotions through emotional contagion and emotional appeal and ultimately affect their job performance. Based on the affective events theory, this study examines the impact of emotional leadership on the subordinates' job performance, the mediating role of subordinates' positive emotions, and the moderating role of susceptibility to positive emotion. Hierarchical regression analysis of 362 valid questionnaires showed that: (1) emotional leadership has a significant positive effect on subordinates' job performance; (2) subordinates' positive emotion partially mediated the relationship between emotional leadership and subordinates' job performance; (3) subordinates' susceptibility to positive emotion positively moderated the relationship between emotional leadership and positive emotions, i.e., the higher the subordinates' susceptibility to positive emotion, the greater the effect of emotional leadership on their positive emotions. This study validates affective events theory, deepens the understanding of the influence mechanism and boundary conditions of emotional leadership on subordinates' job performance, and provides some references for employee performance management.

## Introduction

Global economic turmoil and accelerated competition make companies face severe survival and development challenges, and human resources are strategic resources to win the initiative of competition. Whether employees can complete their work according to quality and quantity, and at the same time actively perform some organizational citizenship behaviors, is crucial to organizational development. Therefore, employee job performance has always been an important research focus in the field of organizational behavior and human resource management.

Job performance refers to behaviors and results that employees show in their work and are closely related to their goals (Rich et al., 2010). Employees' emotions have a significant impact on their job performance (Grobelna, 2019; Moin et al., 2020), such as negative emotions like anger and sadness, can reduce employees' job performance (Rispens and Demerouti, 2016; Saulnier et al., 2020), and positive emotions, such as happiness and hope, can increase performance (Yavas et al., 2013; Im et al., 2018). However, with the acceleration of competition, excessive mental stress and emotional exhaustion have seriously affected employees' performance (Janssen et al., 2020; Tome and van der Vaart, 2020), and it is more prominent for employees in rapidly developing Chinese organizations.

Leadership style is an important antecedent of subordinates' job performance. Previous studies have shown that humble leadership (Cho et al., 2021), ethical leadership (Aftab et al., 2021), and transformational leadership (Hira et al., 2020) can improve employee job performance. According to affective events theory, leadership style often affects employees' job performance by affecting their emotions (Reizer et al., 2019). Integrating leadership research with emotion research has been a major trend in recent years (Humphrey et al., 2016). However, most previous studies focused on the impact of employees' own emotions on their job performance (Kaur and Sukhmani, 2017; Cortini et al., 2019), and a few research focused on the impact of leaders' emotional expressions and emotion regulation behaviors on employee job performance in Chinese organizations. Leadership is an interactive process in which leaders communicate with their subordinates and emotion plays an important role in the interactive process (Gooty et al., 2010). A leader's emotions can influence subordinates through the conscious or unconscious state (Bhullar, 2012), and subordinates' emotions can affect their performance (Brief and Weiss, 2002; Bauer and Spector, 2015). Especially due to the high power distance culture, Chinese employees generally agree with the inequality of power in organizations (Hofstede and Minkov, 2005) and are more receptive to being led and directed, thus Chinese employee behavior is more influenced by leaders than that of Western organizations.

Emotional leadership refers to leaders' effective influence and management of subordinates' emotional states (Kaplan et al., 2014). High-emotional leaders are empathetic, can put themselves in the shoes of employees, and enhance subordinates' positive emotions through emotional contagion or other strategies to influence their work attitudes and behaviors (Thiel et al., 2015). Some studies have pointed out that the relationship between a leader's empathy and subordinates' job performance is weakened in a culture of high power distance (Sadri et al., 2011). However, some meta-analysis shows that in a culture of high collectivism, the emotional intelligence of leaders has a greater impact on subordinates' task performance (Miao et al., 2018); therefore, compared with Western organizations, Chinese organizations with high power distance and high collectivism, there are two opposite views on the impact of emotional leadership on subordinates' performance. However, existing studies rarely examine the impact of emotional leadership on employee performance in the context of Chinese organizations; at the same time, the emotional support of leaders is a valuable resource for employees, which can enhance employees' positive emotions, emotional attachment, and trust and ultimately improve their job performance (Cuyper et al., 2012). In the context of Chinese organizations, leaders have a greater impact on subordinates, and high emotional leadership behaviors are more valuable to employees and are more likely to arouse their positive emotions. However, few studies have conducted empirical tests on this mechanism in the context of Chinese organizations.

In addition, the effect of emotional leadership has certain boundary conditions, but research on moderating variables related to the mechanism of emotional leadership in Chinese organizations is relatively scarce. Leaders enhance subordinates' positive emotions through emotional contagion or other strategies, but emotional contagion varies from person to person (Dezecache et al., 2015). Subordinates with high susceptibility to emotion are more likely to interpret a leader's emotions and are more susceptible to the leader's emotions (Clarkson et al., 2020). Thus, the process by which emotional leadership affects subordinate performance through emotional pathways may be influenced by subordinates' susceptibility to positive emotion.

Therefore, this study explores the impact of emotional leadership on the subordinates' job performance, the mediating role of subordinates' positive emotions, and the moderating role of subordinates' susceptibility to positive emotion based on affective events theory, to enrich emotional leadership effectiveness research results and provide a reference for organizations to improve employees' job performance.

## Research Hypotheses

### The Impact of Emotional Leadership on Subordinates' Job Performance

Humphrey (2002) first introduced “emotional leadership” as an independent leadership style. However, scholars have different opinions on the connotation of this concept. Scholars with a trait viewpoint see it as a trait that enables leaders to understand the emotions of their subordinates (Jin, 2010), or equate it with a leader's emotional intelligence (Hwa and Sook, 2010). However, emotional intelligence places more emphasis on the management of one's own emotions and the ability to use emotional information to guide one's thoughts and actions. Some scholars also emphasize that leaders can indirectly influence employee behavior by regulating their emotional state to influence employees' emotions (Bhullar, 2012). Other scholars view it as a leader's emotional management behaviors toward subordinates and teams (Newman et al., 2009), such as including tactful interaction, caring support, emotional expression, and open communication (Kaplan et al., 2014).

Emotional leadership is a leadership style, which connotes that the leader manages, guides, and regulates the emotions of organization members using unconscious emotional contagion and conscious strategies, creates a suitable emotional atmosphere for the team, and mobilizes the enthusiasm and motivation of the organization members, thus improving the efficiency of work and accomplishing organizational goals.

Affective events theory points out that employees' work attitudes and behaviors are influenced by various events that occur during the work process. These “emotional events” can directly influence employees' work attitudes and behaviors (Weiss and Cropanzano, 1996). The emotions and behaviors of leaders are important working environment factors for employees, thus according to the affective events theory, a leader can affect employees' work behaviors. Studies have found that leaders' emotions have a significant impact on employees' performance (Van Kleef et al., 2010; Wang and Seibert, 2015). Alternatively, emotional leadership is viewed as a leader's emotion management behavior toward subordinates through tactful interactions, caring support, emotional expression, and open communication (Kaplan et al., 2014), and it has been found that transformational leaders can promote subordinates' self-efficacy, optimism, and psychological security and enhance their performance through personalized care and intellectual stimulation (Rowold and Rohmann, 2009). Therefore, leaders with high emotional leadership may improve subordinates' job performance through caring support and open communication. When emotional leadership is defined in terms of leader emotional intelligence, meta-analysis results show that leader emotional intelligence is a significant predictor of subordinate task performance after controlling for the Big Five and cognitive abilities (Miao et al., 2018). Accordingly, the following hypothesis is proposed:

Hypothesis H1: Emotional leadership has a positive effect on subordinates' job performance.

### The Impact of Emotional Leadership on Subordinates' Positive Emotions

Lazarus (1991) pointed out that positive emotions are feelings that occur when an individual achieves a goal or is evaluated positively, while Fredrickson and Branigan (2001) stated that positive emotions are a unique, immediate response to something valuable and include 10 types of emotions, such as joy, hope, pride, interest, humor, serenity, love, motivation, admiration, and gratitude. Affective events theory suggests that work events cause employees to feel emotions accordingly (Weiss and Cropanzano, 1996). Leaders, as important work environment factors for employees, have an impact on employees' emotions. Previous research has found that abusive leaders often express negative emotions and can infect subordinates with negative emotions (Han et al., 2017); while Charismatic leaders often express positive emotional states, which infect subordinates with positive emotions, so subordinates are more likely to feel happy and confident (Sy et al., 2018); furthermore, transformational leaders can promote employees to generate positive emotions through supportive behaviors for employees (Connelly and Ruark, 2010).

Leaders with emotional leadership have strong empathy, are more likely to resonate with their subordinates, understand their subordinates' emotional states (Jin, 2010), infect employees' emotions by regulating their emotional states (Bhullar, 2012), and can enhance subordinates' positive emotions through emotional tactful interaction, caring support, emotional expression, open communication, or other strategies (Kaplan et al., 2014; Thiel et al., 2015), so their subordinates are more likely to feel positive emotions, such as joy, hope, and pride. In addition, leaders' emotional intelligence is significantly positively correlated with employee job satisfaction and positive emotions (Kafetsios and Nezlek, 2011). As mentioned earlier, emotional leadership is also considered by some scholars to approximate the emotional intelligence of leaders. Therefore, leaders with high emotional leadership can increase employee job satisfaction and positive emotions. Accordingly, the following hypothesis is proposed:

Hypothesis H2: Emotional leadership has a positive effect on subordinates' positive emotions.

### The Mediating Role of Subordinates' Positive Emotions

Positive emotions can increase individual motivation and activity. In a positive mood, individuals can maintain positive states and proactive behaviors, easily break through limitations of thinking, and generate new ideas. Then, they are willing to explore new things and actively perform their duties at work, thus increasing work output (Fredrickson and Branigan, 2001). It has been found that positive emotions make employees stay optimistic and enthusiastic, energetic and confident in their work, active in accomplishing goals and tasks, and improve job performance (Ouweneel et al., 2012). Therefore, positive emotions have a significant positive effect on job performance (Yavas et al., 2013; Im et al., 2018; Reizer et al., 2019).

Affective events theory states that specific events largely determine employees' mood and emotional state at work, and employees' mood and emotions at work affect their work attitudes and behaviors (Weiss and Cropanzano, 1996), and then employees' job performance will be consequently affected. When the external environment causes a change in an individual's emotional state, his job performance changes as well (Grobelna, 2019). Previous research has found that transformational leaders can influence subordinates' performance by affecting their positive emotions (Rowold and Rohmann, 2009; Reizer et al., 2019), and that leader emotions affect subordinates' creative tasks by influencing their positive emotions (Visser et al., 2013), and that leader's positive sense of humor can enhance subordinates' positive emotions at work and thus enhance their work engagement (Goswami et al., 2016). High-emotional leaders pay attention to the emotional needs of their subordinates and enhance their positive emotions through emotional contagion or other strategies to influence their work attitudes and behaviors (Kaplan et al., 2014; Thiel et al., 2015). Therefore, emotional leadership has a positive effect on subordinates' job performance. Accordingly, the following hypothesis is proposed:

Hypothesis H3: Subordinates' positive emotions mediate the role between emotional leadership and subordinates' job performance.

### The Moderating Role of Subordinates' Susceptibility to Positive Emotions

Affective events theory further states that individual characteristics play moderating roles between emotional events and emotional responses (Weiss and Cropanzano, 1996), thus emotional reactions to the same emotional event vary across individuals. Subordinate characteristics, such as independence and innovation, can moderate the relationship between transformational leadership and subordinates' work engagement (Zhu et al., 2009).

During the influence of interpersonal interactions on subordinates' emotions, an individual's emotional susceptibility is an important characteristic that determines the magnitude of the influence. Emotional susceptibility is the degree to which an individual can be influenced by his or her emotions when performing cognitive activities. Individuals with high emotional susceptibility are more likely to be influenced by their emotions when performing cognitive activities, as well as by the emotional state of other members of the team (Ilies et al., 2007). Research has found that a procedural justice atmosphere has a stronger impact on individual positive emotions in individuals with high emotional susceptibility (Lin, 2015). Since emotional susceptibility varies from person to person (Dezecache et al., 2015), a leader's emotional state and behaviors have different degrees of influence on subordinates. Subordinates with high emotional susceptibility are more likely to be influenced by leaders' working and family conflicts (Baral and Sampath, 2019). In addition, subordinates' emotional susceptibility can facilitate the positive effects of leader abuse management on subordinates' emotional exhaustion (Wu and Hu, 2009).

Subordinates with high emotional susceptibility can more easily interpret a leader's emotions and feelings and are more likely to be positively influenced by leaders who frequently express positive emotions. Employees with high susceptibility are better able to benefit from the emotional assistance provided by leaders (Kaplan et al., 2014). A recent meta-analysis suggests that subordinates' emotional susceptibility moderates the effects of charismatic leadership and transformational leadership on subordinates' positive emotions (Clarkson et al., 2020).

Based on affective events theory, this study argued that high emotional leadership can infect subordinates with positive emotions, and high positive emotions lead to higher levels of subordinate job performance. However, this process may be moderated by the subordinate's susceptibility to positive emotion. The higher the subordinate's susceptibility to positive emotion in this process, the greater the effect of emotional leadership on the subordinate's positive emotion. Accordingly, this study proposed the following hypothesis:

H4: Subordinates' emotional susceptibility positively moderates the relationship between emotional leadership and positive emotions.

**Figure 1:**
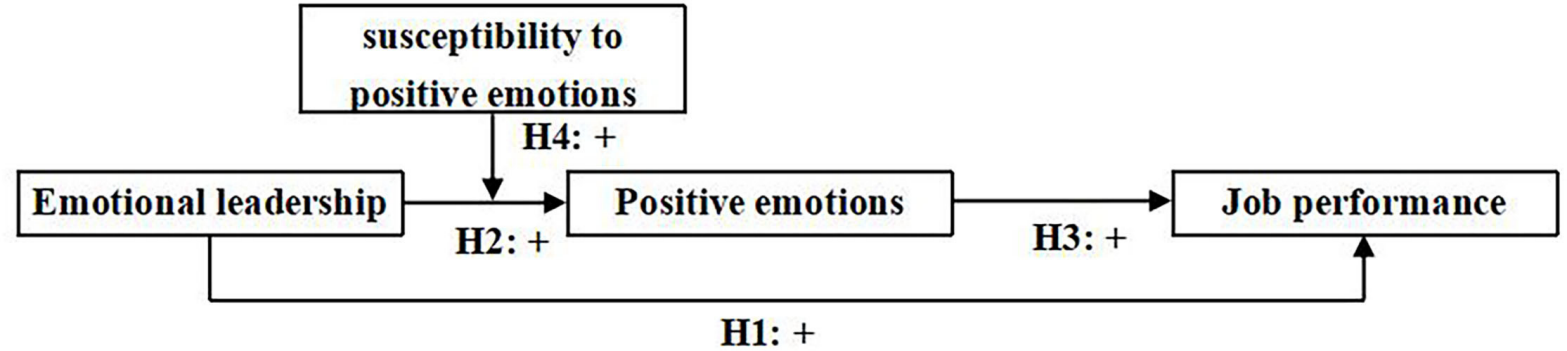
Research model.

Based on affective events theory and broaden and build theory of positive emotions, this study constructs the research model as shown in Figure 1.

## Research Methods

### Research Participants

The participants were all from a machinery manufacturing enterprise in Beijing. Among more than 500 employees of the enterprise, 400 employees were randomly selected and filled in a paper questionnaire. There were 400 paper questionnaires distributed on-site and 393 were collected, of which 362 were valid, with a valid recovery rate of 92.1%.

Among the valid questionnaires, 257 were men, accounting for 71.0%; 105 were women, accounting for 29.0%; 185 were married, accounting for 51.1%; 176 were unmarried, accounting for 48.6%; 1 was divorced or widowed, accounting for 0.3%; 6 had junior school education or below, accounting for 1.7%; 11 had high school or technical secondary school education, accounting for 3.0%; 69 had a junior college education, accounting for 19.1%; 158 had bachelor's degree, accounting for 43.6%; and 118 had master's degree or above, accounting for 32.6%. The mean age of participants was 29.240 years, with a standard deviation of 4.536, and the average time working with current leaders was 3.047 years, with a standard deviation of 2.409.

### Measures

Scales adopted in this study were scored on a 5-point Likert. Notably, 1–5 on the positive emotion scale represent “never” to “always,” respectively, while 1–5 on the other scales represent “strongly disagree” to “strongly agree,” respectively.

Emotional leadership was assessed with Jin's (2010) revised Emotional Competence Scale, which contains seven items, such as “My team leader accurately reads people's moods, feelings or nonverbal cues.” The Cronbach's α in this study was 0.904.

Positive emotions were measured with the positive emotion dimension of the PANAS questionnaire revised by Qiu et al. (2008), which contains nine items, such as “excited” and “grateful.” In this study, Cronbach's α was 0.935.

Job performance was measured using the Task Performance Scale developed by Williams and Anderson (1991), which contains nine items, such as “I always complete the work assigned by the company.” The Cronbach's α in this study was 0.832.

Emotional susceptibility was assessed using the Chinese version of the Emotional Contagion Scale (ECS) revised by Wang et al. (2013), which contains three items, such as “I feel happy when I am with happy people.” In this study, Cronbach's α was 0.803.

## Results

### Common Method Bias Test and Discriminant Validity Analysis

Given that the data were filled in by participants at one time, which may lead to common method bias. First, the Harman one-way test for common method bias was used, and all variable items were subjected to unrotated exploratory factor analysis. The percentage of variance explained by the first factor was 24.714%, which was lower than the 40% criterion. Second, since the Harman one-way test method may be insensitive, the method factor was added as a global factor based on the four-factor model. The four-factor structure of the data fitted well (χ^2^/df = 2.903, root mean square error of approximation (RMSEA) = 0.073, standardized root mean squared error (SRMR) = 0.052, Tucker–Lewis index (TLI) = 0.900, and comparative fit index (CFI) = 0.911), but the model could not be fitted after adding the method factor. Finally, a marker variable that had no theoretical relationship with this study was introduced, the model after adding the marker variable (Bayesian information criterion (BIC) = 21,965.405) was compared with the research model (BIC = 16,573.971), and the research model was significantly better than the model with marker variable. All the above statistical tests showed that there was no serious common method bias in these data.

Discriminant validity analysis performed by confirmatory factor analysis using Mplus in this study revealed that the four-factor structure of the data fitted well (χ^2^/df = 2.903, RMSEA = 0.073, SRMR = 0.052, TLI = 0.900, and CFI = 0.911) and significantly outperformed the three-factor, two-factor, and one-factor models. The factor loadings of all items ranged from 0.547 to 0.857, indicating good discriminant validity of the four variables studied.

### Correlation Analysis

To test the correlation between variables, correlation analysis was conducted using SPSS19.0.

First, as shown in Table 1, there are correlations between gender, age, marriage, education, years of working with the leader, and interaction frequency with the leader per week and subordinates' positive emotions, susceptibility to positive emotion, and job performance. Therefore, gender, age, marriage, education, years of working with the leader, and interaction frequency with the leader per week were used as control variables in this study.

**Table 1:** Correlation coefficient.

**Variable**	***M***	***SD***	**Gender**	**Age**	**Marriage**	**Education**	**Working-age**	**Interaction frequency**	**Emotional leadership**	**Positive emotions**	**Job performance**	**Emotional susceptibility**
Gender	1.290	0.454										
Age	29.240	4.536	−0.051									
Marriage	1.520	0.506	−0.123*	0.514**								
Education	1.980	0.888	−0.106*	−0.027	0.158**							
Working time	3.047	2.409	−0.012	0.345**	0.335**	0.094						
Interaction frequency	3.215	1.110	−0.014	0.022	0.013	−0.087	0.085					
Emotional leadership	3.554	0.709	−0.068	−0.058	−0.043	0.145**	−0.052	0.289**				
Positive emotions	3.262	0.728	−0.027	0.009	−0.014	0.097	0.088	0.224**	0.474**			
Job performance	4.063	0.497	0.016	0.027	0.030	0.106*	0.033	0.154**	0.325**	0.332**		
Emotional susceptibility	4.189	0.590	0.156**	−0.107*	−0.072	−0.019	0.026	0.104*	0.142**	0.113*	0.466**	

*N = 362,*

* *p < 0.05,*

** *p < 0.01,*

*** *p < 0.001, two-tailed test*.

Second, emotional leadership was significantly and positively correlated with subordinates' positive emotions (*r* = 0.474, *p* < 0.01), and it was significantly and positively correlated with subordinates' job performance (*r* = 0.325, *p* < 0.01), and it is higher than the average correlation of 0.27 in low-power distance cultures (Miao et al., 2018); subordinates' positive emotions were significantly and positively correlated with their job performance (*r* = 0.332, *p* < 0.01).

### Main and Mediating Effect Tests

To test each research hypothesis, hierarchical multiple regression analysis was conducted using SPSS19.0. As shown in equation M5 of Table 2, after considering control variables, the positive effect of emotional leadership on subordinates' job performance was significant (β = 0.301, *p* < 0.001), indicating that the higher the level of a leader's emotional leadership, the higher the level of subordinates' job performance. Hypothesis 1 was verified.

**Table 2:** Hierarchical regression analysis.

**Variable**	**Positive emotions**			**Job performance**		
	**M1**	**M2**	**M3**	**M4**	**M5**	**M6**
Gender	−0.0180	0.006	0.001	0.033	0.049	0.048
Age	0.0180	0.023	0.029	0.031	0.034	0.030
Marriage	−0.0730	−0.050	−0.046	−0.004	−0.012	0.023
Education	0.120	0.0390	0.044	0.126*	0.071^+^	0.060^+^
Working-age	0.0750*	0.109*	0.107*	−0.002	0.021^+^	0.002
Interaction frequency	0.228	0.089^+^	0.091^+^	0.165***	0.071	0.052
Emotional leadership		0.448**	0.411**		0.301***	0.207***
Positive emotions						0.210**
Susceptibility to positive emotions			0.058*			
Emotional leadership * emotional susceptibility			0.083+			
*R* ^2^	0.0710	0.246	0.253	0.040	0.119	0.153
Δ*R*^2^	0.0710	0.175	0.007	0.040	0.079	0.034

*N = 362,*

* *p < 0.05,*

** *p < 0.01,*

*** *p < 0.001, two-tailed test*.

In equation M2, after considering control variables, there was a significant positive effect of emotional leadership on subordinates' positive emotions (β = 0.448, *p* < 0.001), indicating that higher levels of leaders' emotional leadership are associated with higher levels of subordinates' positive emotions. Hypothesis 2 was verified.

In equation M6, after considering control variables, both emotional leadership and subordinates' positive emotions have significant positive effects on subordinates' job performance (β = 0.207, *p* < 0.001; β = 0.210, *p* < 0.001), but the β coefficient of emotional leadership on subordinates' job performance decreased compared with equation M5, indicating that subordinates' positive emotions partially mediates the relationship between emotional leadership and subordinates' job performance. Hypothesis 3 was verified.

In equation M3, the interaction term of emotional leadership and subordinates' susceptibility to positive emotions had a significantly positive effect on subordinates' job performance (β = 0.083, *p* < 0.05), indicating that subordinates' susceptibility to positive emotions moderates the effect of emotional leadership on positive emotions. Hypothesis 4 was verified.

According to the path effect decomposition principle, the direct path plays a greater role than the indirect path in the effect of emotional leadership on job performance, and the mediating effect accounts for ~1/3 of the total effect.

### Bootstrap Test

The theoretical model was tested using the Process plug-in of SPSS. Model 7 was chosen, and 5,000 Bootstrap sample analyses revealed a significant mediating effect of positive emotions between emotional leadership and job performance (β = 0.057, CI = [0.023, 0.095]), and hypothesis 3 again was supported. Furthermore, susceptibility to positive emotion moderates the relationship between emotional leadership and positive emotion (β = 0.114, CI = [0.027, 0.316]). However, the moderated mediation model did not pass the test.

**Figure 2:**
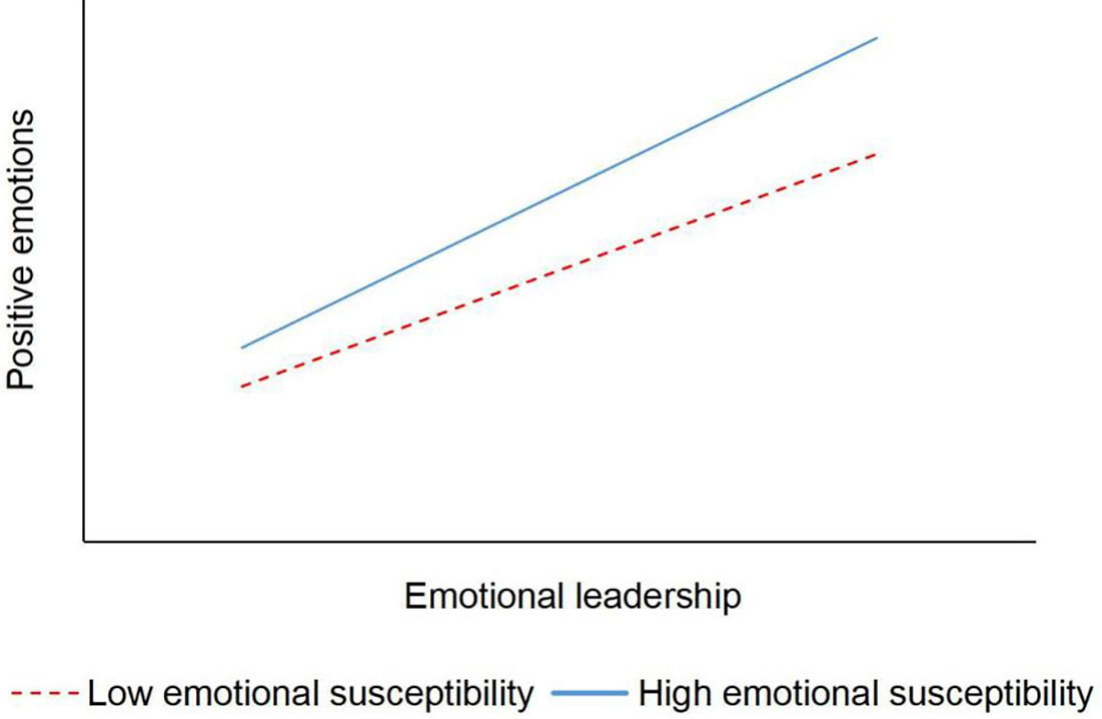
The moderating effect of emotional susceptibility.

A simple slope test was used to analyze the moderating effect of subordinates' susceptibility to positive emotion between emotional leadership and subordinates' positive emotion. Divide high and low groups by the mean of susceptibility to positive emotion plus or minus one standard deviation. As shown in Figure 2, in both high and low susceptibilities to positive emotion groups, emotional leadership had a positive effect on subordinates' positive emotions; in the high susceptibility to the positive emotion group, the positive effect of emotional leadership on positive emotions (β = 0.539, *p* < 0.001) was greater than that of the low group (β = 0.394, *p* < 0.001); and as emotional leadership increased, the positive emotions of the high susceptibility group were higher than those of the low susceptibility group. This indicates that subordinates' susceptibility to positive emotion has an enhancing effect on the relationship between emotional leadership and subordinates' positive emotions. Hypothesis 4 was supported.

## Discussion

### Research Findings

Leaders with emotional leadership have strong empathy, are more likely to resonate with their subordinates, understand their subordinates' emotional states (Jin, 2010), and infect employees' emotions by regulating their emotional states. In addition, the emotions of subordinates can affect their job performance. However, research has focused on the impact of leaders' emotional expressions and emotion regulation behaviors on employee job performance in Chinese organizations. Based on affective events theory, this study examined the impact of emotional leadership on the subordinates' job performance, the mediating role of subordinates' positive emotions, and the moderating role of positive emotional susceptibility. Hierarchical regression analysis of 362 valid questionnaires from Chinese organizations showed that: first, leaders with high emotional leadership can improve employees' emotional states, thus enabling employees to have higher job performance. Second, subordinates' positive emotions partially mediated the relationship between a leader's emotional leadership and subordinates' job performance. Third, subordinates' susceptibility to positive emotion facilitates the relationship between a leader's emotional leadership and subordinates' positive emotions, i.e., the higher the subordinates' susceptibility to positive emotion, the greater the effect of emotional leadership on subordinates' positive emotions.

### Significance

The main theoretical implications of this study are as follows.

First, studies have shown that positive leadership styles, such as humble leadership (Cho et al., 2021), ethical leadership (Aftab et al., 2021), and transformational leadership (Hira et al., 2020), have positive effects on subordinate job performance, such as humble leaders tend to respect and appreciate employees and can make subordinates feel trusted, thereby improving their job performance. However, there is insufficient research on the emotional mechanism of leadership style. In response to the trend of combining leadership research with emotional research (Humphrey et al., 2016), this research proposed, based on affective events theory, that “emotional leadership has positive impacts on subordinates' positive emotions and job performance.” The data obtained from Chinese organizations verify the hypotheses, which provide data support for the cross-cultural effectiveness of emotional leadership. Compared with Western organizations, there are two opposite views on the impact of emotional leadership on subordinates' job performance in Chinese organizations with high power distance and high collectivism (Sadri et al., 2011; Miao et al., 2018). This study used data from Chinese organizations to provide new support for the previous meta-analysis's conclusion that in a culture of high collectivism, the emotional intelligence of leaders has a greater impact on subordinates' job performance.

Second, this study found that emotional leadership has an important impact on subordinates' positive emotions and thus has an important impact on their job performance. It has been found that employees' positive emotions mediate the impact of leadership style on their job performance in Western organizations, such as transformational leaders, can affect their subordinates' job performance by influencing their subordinates' positive emotions (Reizer et al., 2019). This study reconfirms the role of employees' positive emotions in the leadership process in the Chinese context, showing that it has cross-cultural consistency. In addition, this study not only validates the argument of the affective events theory that emotions play a mediating role between external events and employees and behaviors but also shows that the emotional support of leaders is equally or even more a valuable resource for employees in Chinese organizations, which can enhance employees' positive emotions, ultimately improving their job performance. Therefore, it can enrich understanding of the effect mechanism of emotional leadership's on employees' job performance in Chinese organizations.

In addition, this study found that subordinates' susceptibility to positive emotion facilitated the relationship between emotional leadership and subordinates' positive emotions. This result validates the view of affective events theory that individual characteristics play a moderating role between emotional events and individual emotional responses. It is also consistent with conclusions in Western organizations that subordinates with high emotional susceptibility are more likely to interpret the leaders' emotions and are more likely to be affected by leaders' emotions (Clarkson et al., 2020). It showed that there is cross-cultural consistency that employees' emotional susceptibility moderates the relationship between leaders' emotions and employees' emotions.

The findings had the following implications for management practice. First, organizations should focus on assessing the level of emotional leadership when selecting team leaders; or conduct emotional leadership training for leaders to ensure that they can adequately influence employees' positive emotions, including through emotional contagion and other ways. Second, organizations should improve the positive emotions of employees through organizational culture construction, team atmosphere creation, work environment design, etc. Finally, organizations should evaluate the emotional susceptibility of employees; for employees with high emotional susceptibility, leaders could promote their positive emotions by interacting with them, providing caring support, emotional expression, and open communication opportunities.

### Limitations and Prospects

There are some limitations to this study. First, the data were obtained through employee self-assessments, though statistical tests showed that there was no common method bias in the data. Following studies should collect data from multiple data sources to prevent common method bias. Second, the data were collected at a single time point, and subsequent studies should collect data at different time points and use time-delay models to verify the causal relationships. Third, the power distance in Chinese society is relatively high, people in this society generally agree with high power distance relatively. So, we did not regard it as a control variable. In follow-up research, we can treat it as a control variable. Finally, data on emotional leadership were reported by employees, following that research should collect emotional leadership data at the team level and conduct multilevel research.

The following research could provide insight into the following directions. First, this study found that positive emotions partially mediated the relationship between leader emotional leadership and subordinates' job performances. The following research could examine other mediating variables, such as subordinates' internal motivation. Second, the following studies can further investigate whether and how other individual or organizational variables, such as individual traditionalism and organizational positive climate, play moderating roles in the relationship. Third, since the strategies of emotional leadership mainly include emotion recognition, emotion expression, emotion regulation, and emotion contagion, future research can compare the effects of different strategies on subordinates' work performance. In addition, follow-up research can study the impact of other leadership styles, such as inclusive leadership and benevolent leadership on employees' positive emotions and job performance and compare their effects with emotional leadership.

## Author's Note

First, in response to the trend of combining leadership research with emotional research, this research proposes, based on the affective events theory, that “emotional leadership has a positive impact on subordinates' positive emotions and job performance.” The data obtained from Chinese organizations verify the hypothesis, which provides data support for the cross-cultural effectiveness of emotional leadership. So, it provides new support for the previous meta-analysis's conclusion that in a culture of high collectivism, the emotional intelligence of leaders has a greater impact on subordinates' performance.

Second, this study found that emotional leadership can have an important impact on subordinates' positive emotions and thus have an important impact on their job performance. This study not only validates the argument of affective events but also enriches understanding of the effect mechanism of emotional leadership on employees' emotions and behaviors in Chinese organizations.

In addition, this study found that subordinates' susceptibility to positive emotion facilitated the relationship between emotional leadership and subordinates' positive emotions. This result validates the view of affective events theory and also shows that there is cross-cultural consistency that employees' emotional susceptibility moderates the relationship between leadership emotions and employee emotions.

## Data Availability Statement

The raw data supporting the conclusions of this article will be made available by the authors, without undue reservation.

## Ethics Statement

Ethical review and approval was not required for the study on human participants in accordance with the local legislation and institutional requirements. Written informed consent for participation was not required for this study in accordance with the national legislation and the institutional requirements.

## Author Contributions

JW contributed to conceptualization, methodology, data creation, software, and writing of the manuscript. KP contributed to validation and writing—review and editing of the manuscript. YP performed translation, chart-making, and table-making. LM contributed to design questionnaire, questionnaire collection, and questionnaire sorting. All authors contributed to this study and approved the submitted version.

## Funding

This research was supported by the National Natural Science Foundation of China (72161014).

## Conflict of Interest

The authors declare that the research was conducted in the absence of any commercial or financial relationships that could be construed as a potential conflict of interest.

## Publisher's Note

All claims expressed in this article are solely those of the authors and do not necessarily represent those of their affiliated organizations, or those of the publisher, the editors and the reviewers. Any product that may be evaluated in this article, or claim that may be made by its manufacturer, is not guaranteed or endorsed by the publisher.
